# Increased Epoxyeicosatrienoic Acids and Hydroxyeicosatetraenoic Acids After Treatment of Iodide Intake Adjustment and 1,25-Dihydroxy-Vitamin D_3_ Supplementation in High Iodide Intake–Induced Hypothyroid Offspring Rats

**DOI:** 10.3389/fphys.2021.669652

**Published:** 2021-07-26

**Authors:** Qing Liu, Yue Zhang, Hailing Zhao, Xiaomei Yao

**Affiliations:** ^1^Department of Physiology and Pathophysiology, School of Basic Medical Sciences, Tianjin Medical University, Tianjin, China; ^2^Tianjin Key Laboratory of Ionic-Molecular of Cardiovascular Disease, Department of Cardiology, Tianjin Institute of Cardiology, the Second Hospital of Tianjin Medical University, Tianjin, China

**Keywords:** hypothyroidism, 1,25(OH)_2_D_3_, cardiac dysfunction, hypertension, dyslipidemia, iodide intake adjustment, fatty acids

## Abstract

**Aim:** This study aimed to investigate the potential role of fatty acids in high iodide intake–induced hypothyroidism and its complications and also in the intervention of iodide intake adjustment and 1,25-dihydroxy-vitamin D_3_ [1,25(OH)_2_D_3_] supplementation.

**Methods:** Pregnant rats were allocated to two groups, namely, normal iodide (NI, 7.5 μg/day) intake and 100 times higher-than-normal iodide (100 HI, 750 μg/day) intake. The offspring were continuously administered potassium iodide from weaning [i.e., postnatal day 21 (PN21)] to PN90. After PN90, the offspring were either administered iodide intake adjustment (7.5 μg/day) or 1,25(OH)_2_D_3_ supplementation (5 μg·kg^−1^·day^−1^), or both, for 4 weeks. Thyroid function tests (free triiodothyronine, free thyroxine, thyrotropin, thyroid peroxidase antibody, and thyroglobulin antibody), blood lipids (triglyceride, total cholesterol, free fatty acid, and low-density lipoprotein cholesterol), and vitamin D3 (VD3) levels were detected by ELISA. Cardiac function was measured by echocardiography. Blood pressure was measured using a non-invasive tail-cuff system. The serum fatty acids profile was analyzed by liquid chromatography–mass spectrometry.

**Results:** In the offspring rats with continued 100 HI administration, the levels of 8,9-dihydroxyeicosatrienoic acid (8,9-DHET) and thromboxane B2 (TXB2) were decreased, while those of prostaglandin J2 (PGJ2), prostaglandin B2 (PGB2), 4-hydroxydocosahexaenoic acid (4-HDoHE), 7-HDoHE, 8-HDoHE, and 20-HDoHE were increased. Significant correlations were found between PGB2, 8,9-DHET, 7-HDoHE levels and thyroid dysfunction, between PGJ2, 20-HDoHE, PGB2, 8,9-DHET levels and cardiac dysfunction, between PGJ2, 20-HDoHE levels and hypertension, between 4-HDoHE, 8-HDoHE, TXB2 levels and dyslipidemia, and between PGB2 and decreased VD3 level. After the treatment of iodide intake adjustment and 1,25(OH)_2_D_3_ supplementation, the levels of 16-hydroxyeicosatetraenoic acids (16-HETE), 18-HETE, 5,6-epoxyeicosatrienoic acid (5,6-EET), 8,9-EET, 11,12-EET, 14,15-EET, PGE2, 5-oxo-ETE, and 15-oxo-ETE were increased. The significant associations between PGE2, 16-HETE, 18-HETE and improved thyroid function and also between 5,6-EET, 11,12-EET, 14,15-EET, 16-HETE, 15-oxo-ETE and attenuated dyslipidemia were detected.

**Conclusion:** Increased levels of prostaglandins (PGs) and HDoHEs and decreased levels of 8,9-DHET and TXB2 might occur in the progression of cardiac dysfunction, hypertension, and dyslipidemia in high iodide intake–induced hypothyroidism. The increased levels of EETs and HETEs might help to ameliorate these complications after iodide intake adjustment and 1,25(OH)_2_D_3_ supplementation.

## Introduction

Excess iodine consumption may lead to hypothyroidism (Bürgi, 2010), hyperthyroidism (Roti and Uberti, 2001), and autoimmune thyroid diseases (Laurberg et al., 2010). Serrano-Nascimento et al. investigated the effects of administering five times higher-than-normal iodide (5 HI) [i.e., sodium iodide (NaI)] during the pregnancy and lactation period of rats. The results showed hypothyroidism with the decreased circulating levels of free triiodothyronine (FT3) and free thyroxine (FT4) in offspring at postnatal day 90 (PN90) (Serrano-Nascimento et al., 2017). Our study has shown that 100 HI [i.e., potassium iodide (KI)] during the pregnancy and lactation period of rats can induce the decrease of FT3, FT4, and vitamin D3 (VD3) and also the increase of thyroid peroxidase antibody (TPOAb) and thyroglobulin antibody (TgAb) levels in offspring at PN120. In addition, we demonstrated the protective effect of iodide intake adjustment, 1,25-dihydroxy-vitamin D_3_ [1,25(OH)_2_D_3_] supplementation, or both in offspring rats following excess iodide intake (Wang et al., 2020). Hypothyroidism is a commonly encountered clinical condition, and it can impact cardiac function (Klein and Danzi, 2007; Udovcic et al., 2017), blood pressure (Klein and Danzi, 2007), lipid parameters (Jabbar et al., 2017), and vitamin D level (Salma et al., 2020).

**Figure 1:**
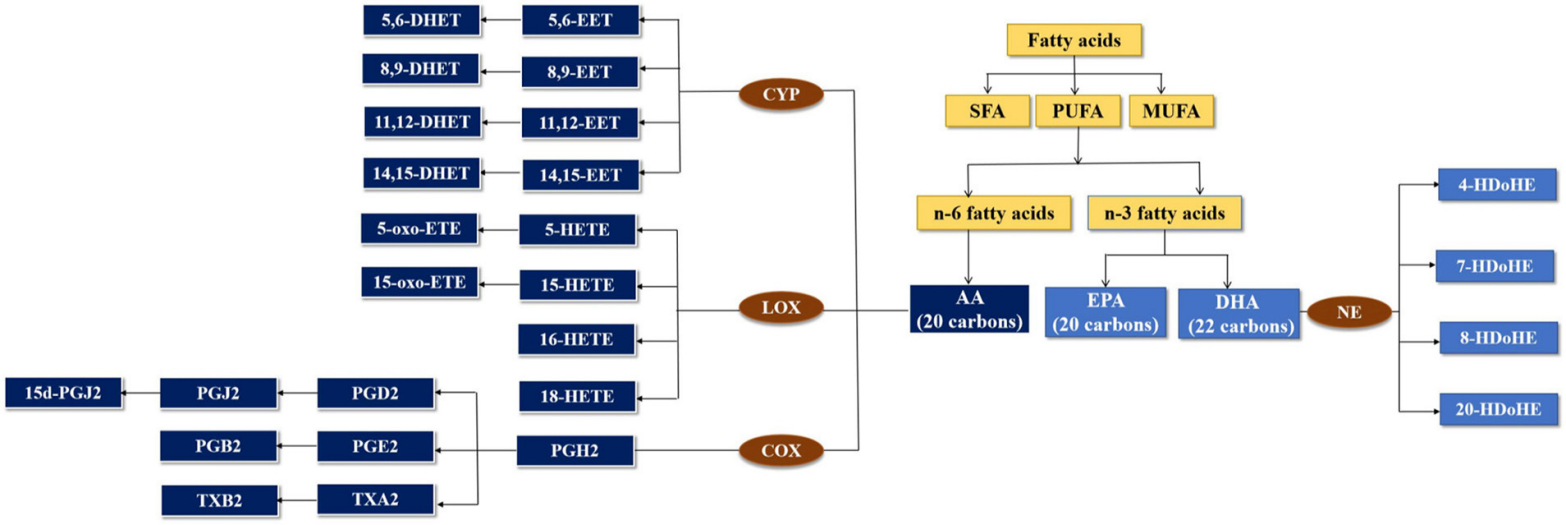
Metabolism of fatty acids by cytochrome P450 (CYP), lipoxygenase (LOX), and cyclooxygenase (COX) enzymes.

Fatty acids occur in the form of mixtures of saturated fatty acid (SFA), monounsaturated fatty acid (MUFA), and polyunsaturated fatty acid (PUFA) (Chen and Liu, 2020). PUFA can be classified into n-3 fatty acids and n-6 fatty acids. Arachidonic acid (AA) is synthesized from the n-6 fatty acid. Eicosapentaenoic acid (EPA) and docosahexaenoic acid (DHA) are synthesized from the n-3 fatty acid (Coras et al., 2021). AA and EPA were 20 carbons in the chain, and DHA was 22 carbons in the chain. Cyclooxygenase (COX) is the oxidase in the pathway for producing prostaglandin (PG) and thromboxane (TX) from AA. Lipoxygenase (LOX) is responsible for producing hydroxyeicosatetraenoic acids (HETEs). Cytochrome P450 (CYP) metabolizes AA to epoxyeicosatrienoic acids (EETs). DHA was metabolized by non-enzymatic (NE) (Figure 1).

Epoxyeicosatrienoic acids (EETs), the CYP epoxygenase metabolites of AA (Neckár et al., 2019), can prevent various cardiovascular diseases (CVDs) and can modulate cardiac and vascular physiology to maintain cardiovascular homeostasis, due to their vasodilator, antihypertensive, and other beneficial biological actions (Imig, 2012). PGs and TXs from AA have been associated with CVD (Dasilva and Medina, 2019), hypertension (Cavalca et al., 2010), and hypercholesterolemia (Davi et al., 1997).

However, the studies to determine the profile of fatty acids in hypothyroidism are scarce. The formation of fatty acids, their role in high iodide intake–induced hypothyroidism and its related complications, and the impact of iodide intake adjustment and 1,25(OH)_2_D_3_ supplementation on the modulation of fatty acid synthesis remain unclear. The application of fatty acids as a therapeutic option for high iodide intake–induced hypothyroidism and its complications may be promising.

## Materials and Methods

### Animals and Administration

**Figure 2:**
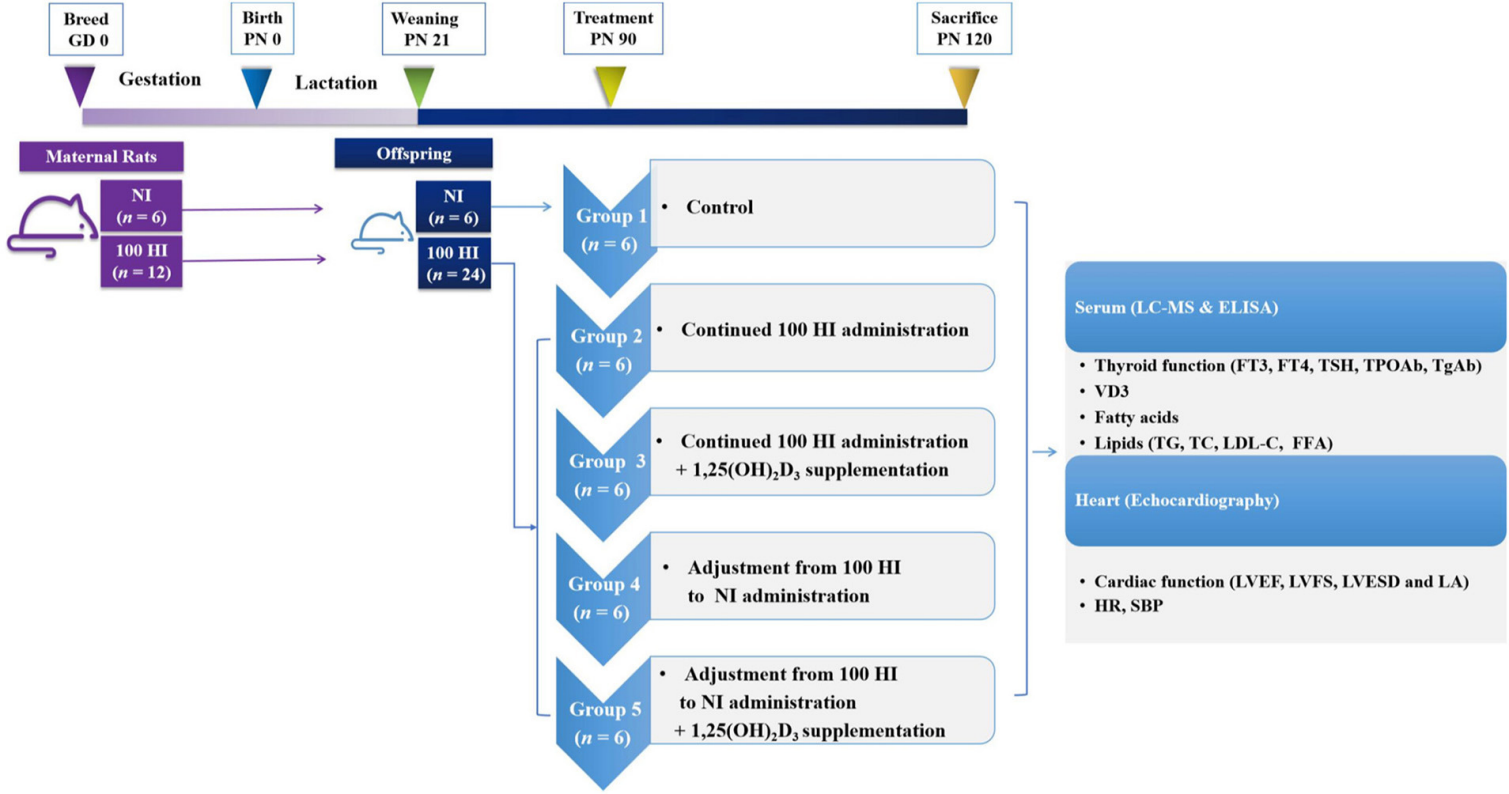
Study plan and timing.

Healthy Wistar rats (Beijing Vital River Laboratory Animal Technology Co., Ltd., Beijing, China) were housed in the specific pathogen-free (SPF) level of the Experimental Animal Center of Tianjin Medical University. In this study, 7-week-old female Wistar rats were mated with fertile males (1:1). The presence of a vaginal plug or sperm in the vaginal smear of the female rats was indicative of pregnancy (Day 0 of gestation, GD 0). The pregnant rats were randomly assigned to two groups, namely, normal iodide (NI) intake (*n* = 6) and 100 HI intake (*n* = 12). The offspring were continuously administered potassium iodide (KI) from weaning [i.e., postnatal day 21 (PN21)] to PN90. After PN90, the rats with NI were held as the control group (group 1, *n* = 6), and the rats with 100 HI were randomly divided into four treatment groups, namely, continued 100 HI administration (group 2, *n* = 6), continued 100 HI administration + 1,25(OH)_2_D_3_ supplementation (group 3, *n* = 6), adjustment from 100 HI to NI administration (group 4, *n* = 6), and adjustment from 100 HI to NI administration + 1,25(OH)_2_D_3_ supplementation (group 5, *n* = 6) (Figure 2). The rats in the NI group received dietary feed containing iodide (7.5 μg/day), in addition to the oral administration of deionized water. The rats in the 100 HI group received deionized water containing KI (24,750 μg/L) and dietary iodide. Therefore, the intake of iodide was 750 μg/day (Wang et al., 2018). The rats in groups 3 and 5 were supplemented with 1,25(OH)_2_D_3_ (MedChemExpress, Monmouth Junction, NJ, USA) by gavage (5 μg·kg^−1^·day^−1^) (Wang et al., 2020). The animal study was reviewed and approved by the Institutional Animal Care and Use Committee of Tianjin Medical University (No. TMUaMEC 2016054).

### Measurements of Thyroid Function, Lipid, and VD3 Levels in Serum by ELISA

Blood samples were drawn from the orbital sinus, centrifuged, and stored at −80°C. The levels of FT3, FT4 (Meilian Biological Technology, Shanghai, China), thyrotropin (TSH) (ImmunoWay Biotechnology Company, TX, USA), TPOAb, and TgAb (MyBioSource, San Diego, CA, USA), the lipid levels of total cholesterol (TC), triglyceride (TG), low-density lipoprotein cholesterol (LDL-C), and free fatty acid (FFA) (Nanjing Jiancheng Bioengineering Institute, China), and the VD3 level (Meilian Biological Technology, Shanghai, China) in the serum were determined by the rat-specific ELISA kits.

### Analysis of Fatty Acids in Serum by Liquid Chromatography–Mass Spectrometry (LC–MS)

Serum was extracted by solid-phase extraction (SPE) as described by Zhang X. et al. (2015). A UPLC BEH C18 column (1.7 μm, 100 × 2.1 mm i.d.; Waters, Milford, MA, USA) consisting of ethylene-bridged hybrid particles (Waters, Milford, MA, USA) was used for chromatographic separations. Solvent A was water, and solvent B was acetonitrile. The mobile-phase flow rate was 0.6 mL/min (column temperature, 25°C). The injection volume was set to 10 μl. A total of 27 AA and 24 n-3 PUFA metabolites were profiled by multiple reaction monitoring (MRM) scans in negative mode, which involved the use of a 5,500 QTRAP hybrid triple quadrupole linear ion-trap mass spectrometer (AB Sciex, Foster City, CA, USA) equipped with a turbo ion-spray electrospray ionization source. The ion source parameters were as follows: CUR = 40 psi, GS1 = 30 psi, GS2 = 30 psi, IS = −4,500 V, CAD = medium, and temperature = 500°C.

### Cardiac Function Measured by Echocardiography

The rats were anesthetized with inhaled isoflurane and fixed onto the operation table, and the probe was placed on the left chest. The M-mode images were obtained from the short axis of the left ventricle at the level of the papillary muscle. A VisualSonics echocardiographic system equipped with a 30-MHz transducer (RMV-707B, Toronto, Canada) and the Vevo2100 version 3.0.0 software (VisualSonics Inc., Canada) was used.

### Measurement of Systolic Blood Pressure

The rats were trained to become familiar with the restrainer of the rat and to remain calm during the monitoring of blood pressure. The systolic blood pressure (SBP) for each rat was monitored by the “non-invasive tail-cuff system” (Visitech, BP-2000 Series II, Ohio, USA). The SBP measurement was always carried out in the afternoon, and the data were obtained from the average of measuring three times.

### Statistical Analysis

Fatty acids were quantified with the use of MultiQuant 2.1 software (AB Sciex, Foster City, CA, USA). Fold change (FC) >2 and *p*-value < 0.05 represent a significant result (Shaker et al., 2020). Metaboanalyst 3.0 (http://www.metaboanalyst.ca) was used for the metabolomic data analysis, interpretation, and visualization, which is presented as a heat map and a volcano plot (Xia et al., 2015). The data were compared and analyzed using IBM SPSS Statistics for Windows Version 22.0 (IBM Corp. Armonk, NY, USA). The control group and the continued 100 HI administration group were compared by using the independent samples *t*-test. The normal distribution of continuous variables was verified using the Kolmogorov–Smirnov test (*p* < 0.05). The two-way ANOVA was used to analyze the effects of treatment with iodide intake adjustment and/or 1,25(OH)_2_D_3_ supplementation. Pearson's correlations were used to detect the relationship between fatty acids, thyroid function, VD3, lipids, and parameters of cardiac function. The data were expressed as the mean ± SD and *n* = 6 for each group. The *p* < 0.05 was considered significant.

## Results

### No Significant Change in Body Weight Following Iodide Adjustment and/or 1,25(OH)_2_D_3_ Supplementation for 4 Weeks

The body weight of offspring rats at PN120 per group were as follows: 329.8 ± 63.27 g (group 1, *n* = 6), 301.11 ± 75.15 g (group 2, *n* = 6), 309.68 ± 87.29 g (group 3, *n* = 6), 303.93 ± 60.76 g (group 4, *n* = 6), and 301.22 ± 64.27 g (group 5, *n* = 6). There was no significant difference in body weight among the five groups (*p* > 0.05).

### Fatty Acids Profile in the Continued 100 HI Administration Group

**Figure 3:**
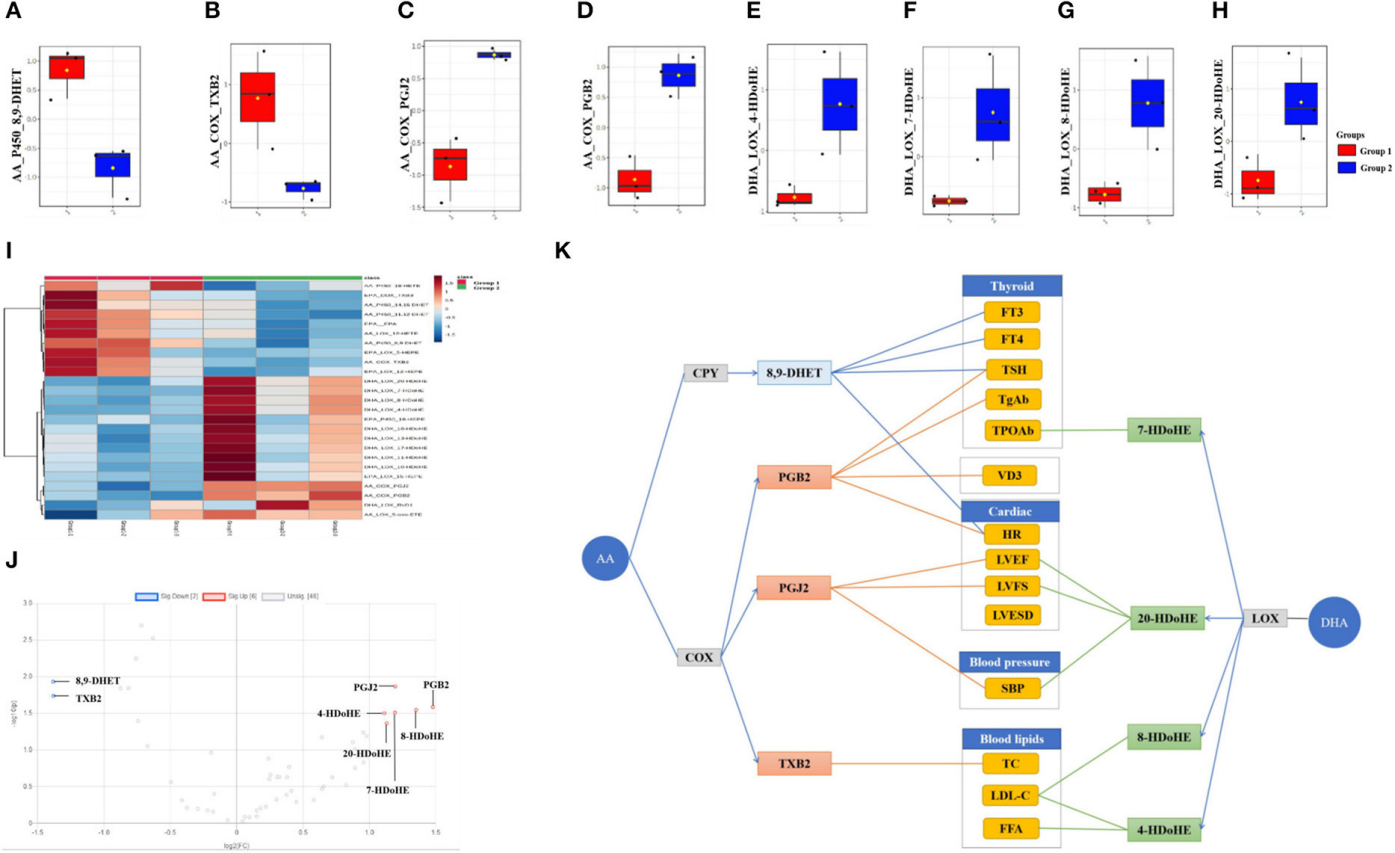
Fatty acids profile and correlation analysis in the continued 100 HI administration group (group 2). **(A–H)** Box plots of the fatty acids levels. The bar in the quartile-indicating box is the median value. **(I)** Heat map shows the fatty acids profile from group 1 and group 2. **(J)** Volcano plot shows the significance and fold change in expression of fatty acids. **(K)** Correlation analysis between significantly changed fatty acids and thyroid function, cardiac function, blood lipids, and vitamin D3 (VD3) levels in the continued 100 HI administration group (group 2). *n* = 6 for each group.

The top 25 fatty acids were visualized in a heat map, which enabled effective differentiation between the control group (group 1) and the continued 100 HI administration group (group 2) (Figure 3I). The upregulation or downregulation with significance was identified in a volcano plot, which was compared with the log2-FC of the levels of fatty acids of significance (log *t*-test). In the continued 100 HI administration group (group 2), six fatty acids were significantly upregulated, including two fatty acids derived from AA: PGB2, PGJ2; four fatty acids derived from DHA: 4-hydroxydocosahexaenoic acids (HDoHE), 7-HDoHE, 8-HDoHE, and 20-HDoHE, while another two fatty acids derived from AA were significantly downregulated, namely, 8,9-dihydroxyeicosatrienoic acid (DHET) and TXB2 (Figure 3J). The median levels of 8,9-DHET and TXB2 were significantly decreased, while those of PGJ2, PGB2, 4-HDoHE, 7-HDoHE, 8-HDoHE, and 20-HDoHE were significantly increased in the continued 100 HI administration group (group 2) when compared with the control group (group 1). No significant alteration was detected in other fatty acids (Figures 3A–H).

### Effects of Fatty Acids With Significant Change on Thyroid Function, Cardiac Function, Blood Lipids, and VD3 Levels in the Continued 100 HI Administration Group

**Figure 4:**
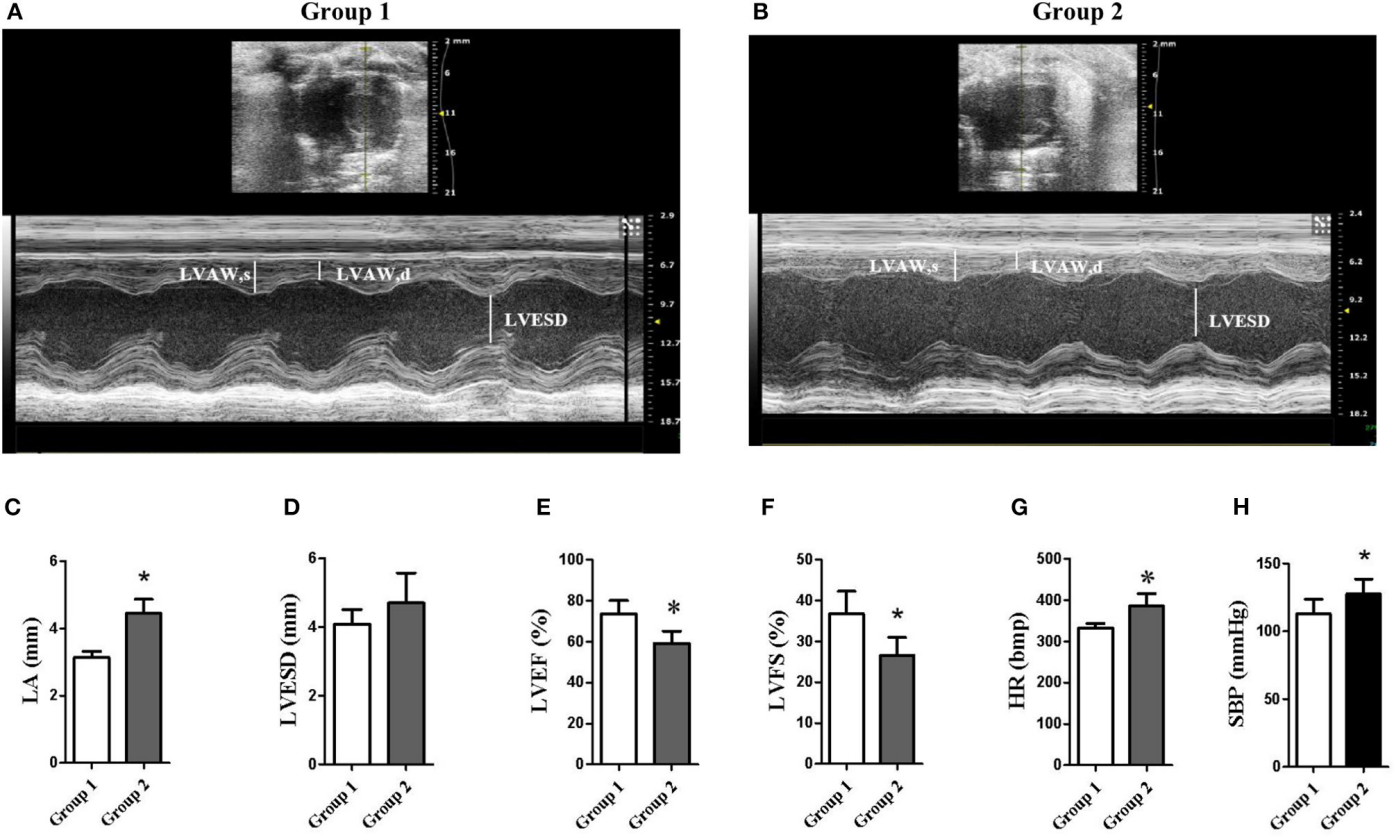
Echocardiography data. **(A,B)** Representative examples of M-mode and B-mode echocardiographic tracings from group 1 (control) and group 2 (continued 100 HI administration). **(C–G)** Measurements were assessed by the echocardiographic data. *p < 0.05. **(H)** Systolic blood pressure (SBP). LVAW, left ventricular anterior wall. *n* = 6 for each group.

Compared with the control group (group 1), FT3, FT4, and VD3 levels were significantly decreased, TSH, TPOAb, and TgAb levels were significantly increased, and blood lipid levels (i.e., TG, TC, FFA, and LDL-C) were also significantly increased in the continued 100 HI administration group (group 2) (*p* < 0.05) (Table 1). Although there was no significant change in the left ventricular end-systolic diameter (LVESD), the left ventricular ejection fraction (LVEF) and the left ventricular fractional shortening (LVFS) were significantly reduced, and the left atrial (LA) dimension and the heart rate (HR) were significantly increased in cardiac function measured by echocardiography. For blood pressure, SBP was significantly increased (Figure 4).

**Table 1:** Thyroid hormone, autoantibody, blood lipids, and vitamin D3 (VD3) levels in different treatment groups.

**Groups**	**Thyroid hormone and autoantibody**					**Blood lipids and VD3**				
	**FT3 (pmol/L)**	**FT4 (pmol/L)**	**TSH (ng/mL)**	**TPOAb (IU/mL)**	**TgAb (ng/L)**	**TG (mmol/L)**	**TC (mmol/L)**	**FFA (mmol/L)**	**LDL-C (mmol/L)**	**VD3 (ng/mL)**
Group 1	8.75 ± 0.46	12.75 ± 1.40	9.67 ± 0.95	47.30 ± 1.60	10.95 ± 1.11	0.70 ± 0.07	2.59 ± 0.20	2.59 ± 0.20	1.20 ± 0.12	26.62 ± 1.74
Group 2	7.54 ± 0.30^*^	9.85 ± 1.17^*^	12.53 ± 0.44^*^	59.58 ± 5.73^*^	18.52 ± 2.26^*^	1.26 ± 0.05^*^	4.68 ± 0.15^*^	4.68 ± 0.15^*^	2.09 ± 0.37^*^	14.27 ± 0.74^*^
Group 3	9.48 ± 0.59^#^	11.80 ± 0.85^#^	14.22 ± 2.30	50.46 ± 5.94	18.19 ± 1.50	0.84 ± 0.05^#^	3.28 ± 0.20^#^	3.28 ± 0.20^#^	1.47 ± 0.18^#^	17.62 ± 0.71^#^
Group 4	9.45 ± 0.80^#^	11.58 ± 0.18^#^	12.73 ± 1.23	49.48 ± 3.89	17.12 ± 2.87	0.96 ± 0.05^#^	3.61 ± 0.14^#^	3.61 ± 0.14^#^	1.39 ± 0.09^#^	17.85 ± 1.89^#^
Group 5	9.10 ± 0.69^#^	12.90 ± 0.37	14.47 ± 0.81	57.35 ± 6.27	15.73 ± 1.18	0.88 ± 0.05^#^	2.98 ± 0.21^#^	2.98 ± 0.21^#^	1.36 ± 0.17^#^	18.30 ± 1.53^#^

*Group 1, control; Group 2, continued 100 HI administration; Group 3, continued 100 HI administration + 1,25(OH)_2_D_3_ supplementation; Group 4, adjustment from 100 HI to NI administration; Group 5, adjustment from 100 HI to NI administration + 1,25(OH)_2_D_3_ supplementation*.

* *p < 0.05 vs. group 1*.

# *p < 0.05 vs. group 2. n = 6 for each group*.

The correlation analysis was used to investigate the effect of metabolites with significant change. We showed that thyroid function was correlated with PGB2, 8,9-DHET, and 7-HDoHE, cardiac function was correlated with PGB2, PGJ2, and 8,9-DHET, blood pressure was correlated with PGJ2 and 20-HDoHE, blood lipid was correlated with 4-HDoHE, 8-HDoHE, and TXB2, and VD3 was correlated with PGB2 (Figure 3K).

### Alteration of Fatty Acids in Different Treatment Groups

**Figure 5:**
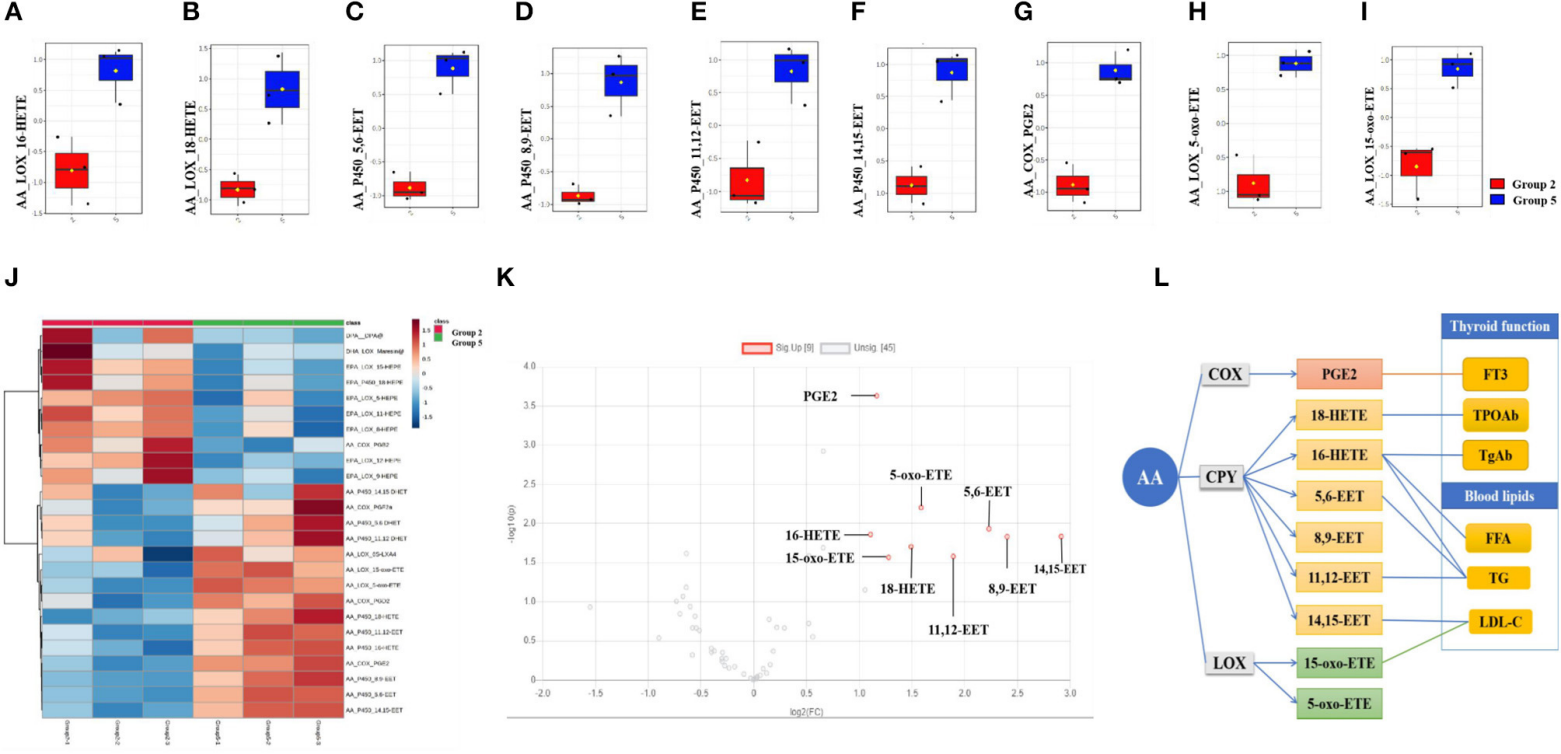
Fatty acids profile and correlation analysis in adjustment from 100 HI to NI administration +1,25(OH)_2_D_3_ supplementation group (group 5). **(A–I)** Box plots of the fatty acids levels in serum. The bar in the quartile-indicating box is the median value. **(J)** Heat map from group 5 and group 2. **(K)** Volcano plot. **(L)** Correlation analysis. *n* = 6 for each group.

Compared with the continued 100 HI administration group (group 2), although no significant alteration was detected in both the continued 100 HI administration + 1,25(OH)_2_D_3_ supplementation group (group 3) and the adjustment from 100 HI to NI administration group (group 4), nine significantly upregulated fatty acids were determined in the adjustment from 100 HI to NI administration + 1,25(OH)_2_D_3_ supplementation group (group 5) in a volcano plot (Figures 5J,K). The median levels of 16-HETE, 18-HETE, 5,6-EET, 8,9-EET, 11,12-EET, 14,15-EET, PGE2, 5-oxo-ETE, and 15-oxo-ETE were significantly increased in the adjustment from 100 HI to NI administration + 1,25(OH)_2_D_3_ supplementation group (group 5) in box plots (Figures 5A–I).

### Effects of Significantly Changed Fatty Acids in the Adjustment From 100 HI to NI Administration + 1,25(OH)_2_D_3_ Supplementation Group

Compared with the continued 100 HI administration group, the levels of FT3 and VD3 were significantly increased, and the levels of blood lipids (i.e., TG, TC, FFA, and LDL-C) were significantly decreased in the continued 100 HI administration + 1,25(OH)_2_D_3_ supplementation (group 3), the adjustment from 100 HI to NI administration (group 4), and the adjustment from 100 HI to NI administration + 1,25(OH)_2_D_3_ supplementation (group 5) (*p* < 0.05) (Table 1).

In the adjustment from 100 HI to NI administration + 1,25(OH)_2_D_3_ supplementation group (group 5), thyroid function correlated with PGE2, 16-HETE, and 18-HETE, and blood lipid correlated with 5,6-EET, 11,12-EET, 14,15-EET, 16-HETE, and 15-oxo-ETE (Figure 5L).

## Discussion

Our findings reported in this study showed that 100 HI iodide intake during the pregnancy and lactation period of rats can induce hypothyroidism with decreased FT3 and FT4 levels and increased TSH levels, resulting in the complication of cardiac dysfunction in offspring rats at PN120. Significant correlations were found between PGJ2, 20-HDoHE, PGB2, 8,9-DHET, and cardiac dysfunction. Triiodothyronine (T3) is the main regulator of gene expression in myocardial muscle, and the decreased T3 in hypothyroidism can affect myocardial contractility and remodeling (Kahaly and Dillmann, 2005; Udovcic et al., 2017). Hypothyroidism is related to cardiac dysfunction. Bassel et al. (2007) reported that, in propylthiouracil (PTU)-induced hypothyroidism rats, LVEF and LVFS were decreased significantly. Fu et al. (2017) reported that, in children with chronic viral myocarditis complicated with arrhythmia, the significantly decreased levels of FT3 and FT4 and the significantly increased levels of TSH, TPOAb, and TgAb were the independent risk factors of malignant arrhythmia. Ren et al. (2019) demonstrated that, in 2,663 euthyroid individuals, SBP was positively correlated with TSH and was an independent predictor of the serum TSH levels. The increased PGB2 and the decreased 8,9-DHET were correlated with HR, the increased PGJ2 was correlated with LVEF and LVFS, and the increased LA and HR, the increased HDoHEs (4-HDoHE, 7-HDoHE, 8-HDoHE, and 20-HDoHE), and the decreased TXB2 were also observed in high iodide intake–induced hypothyroidism. DHETs are the metabolites of EETs hydrolyzed by soluble epoxide hydrolase (sEH) (Yang et al., 2013). PGB2 is the metabolite of PGE2 (Coras et al., 2021), and PGE2 can promote cell growth and elevate the expression of hypertrophic marker genes by inhibiting the COX2/PGE2 pathway (Zhang et al., 2019). PGJ2 is formed by dehydration within the cyclopentenone ring of PGD2 (Abdelrahman et al., 2004). Stelling et al. (2020) reported that the PGD2 level was significantly increased in male mice cardiomyocytes with a cardiomyocyte-specific transcription factor-3 (STAT3) deficiency conditional knockout (CKO), which is an impairment of the endogenous cardiac regeneration potential as it shifts the differentiation potential of the cardiac progenitor cell (CPC) pool from endothelial cells toward white adipocytes, thereby promoting heart failure, a condition that impairs androgen receptor (AR) signaling in the absence of STAT3, which reduces the expression of the PG-degrading enzyme 15-hydroxyprostaglandin-dehydrogenase (HPGD). HDoHEs are biosynthesized from DHA, and the function is still not clearly elucidated. Reynaud et al. (1993) reported that 20-HDoHE was increased during the early period of oxidative stress *in vitro*, indicating that it probably played a pro-inflammatory role. Yao et al. (2015) reported that the serum TXB2 level was significantly elevated in patients with hyperthyroidism. Following the treatment with iodide intake adjustment + 1,25(OH)_2_D_3_ supplementation, 5,6-EET, 8,9-EET, 11,12-EET, and 14,15-EET levels were significantly increased. Although there was no significant change detected for PGB2, a downward trend was apparent. The heart can metabolize EETs, which in turn may play an important role in modulating the electrophysiological properties of the heart. Lee et al. (1999) reported that 8,9-EET and the other EET regioisomers are the potent voltage-dependent inhibitors of the cardiac Na^+^ channels. Na^+^ channel-blocking drugs are the most commonly used pharmacological agents for the treatment of arrhythmias. 11,12-EET has been shown to enhance the recovery of cardiac function following global ischemia (Wu et al., 1997). Neckár et al. (2019) demonstrated that 2-week oral treatment with the EET analog EET-B improved cardiac function in spontaneously hypertensive rats with congestive heart failure induced by myocardial infarction. This study reveals that the increased EETs may serve to alleviate cardiac dysfunction associated with high iodide intake–induced hypothyroidism.

It was reported that there is some relationship between metabolites of AA in both serum and the heart. Hu et al. (2017) reported that 46 and 50 eicosanoids were detected and quantified in the plasma and heart tissue, respectively, and 43 overlapped eicosanoids were detected in plasma and the heart. The vast majority of plasma and heart tissue eicosanoids presented positive correlations with each other. In present study, 8,9-DHET, PGB2, 8-HDoHE, 20-HDoHE, 5,6-EET, 8,9-EET, 11,12-EET, 16-HETE, 18-HETE, and 15-oxo-ETE are among the overlapped eicosanoids. Al-Lawati et al. (2020) reported that, in adjuvant arthritis rats, the changes of total EET concentration in both the plasma and the heart were parallel to each other. The concentrations of the cardioprotective 8,9-EET, 11,12-EET, and 14,15-EET were reported to be parallel to each other in the plasma as well as in the heart (Theken et al., 2011; Aghazadeh-Habashi et al., 2018). In this study, although we did not measure the contents in the heart, according to the previous studies, the changes of cardioprotective 8,9-EET, 11,12-EET, and 14,15-EET in serum may be parallel with those in the heart.

Besides, in our experiment, blood pressure was significantly increased in high iodide intake–induced hypothyroidism. Significant correlations were found between PGJ2, 20-HDoHE, and hypertension. It is well known that thyroid hormones can impact the renin-angiotensin-aldosterone system, and the renin substrates are synthesized in the liver under the stimulus of T3, which results in hypertension in a hypothyroid state (Klein and Danzi, 2007). Iqbal et al. (2006) reported that both the systolic and the diastolic blood pressure were elevated in hypothyroidism. We have shown that the increased PGJ2 was correlated with blood pressure in high iodide intake–induced hypothyroidism. PGJ2 is formed by dehydration within the cyclopentenone ring of PGD2 (Abdelrahman et al., 2004). PGD2 is a vasoconstrictor (Ogletree, 1982). Asirvatham-Jeyaraj et al. (2019) reported that the PGD2 level was increased during the developmental stage of angiotensin II-salt hypertension in Sprague–Dawley rats. We found that EETs (i.e., 5,6-EET, 8,9-EET, 11,12-EET, and 14,15-EET) and HETEs (i.e., 16-HETE and 18-HETE) levels were significantly increased after the treatment of iodide intake adjustment + 1,25(OH)_2_D_3_ supplementation. These findings suggest that the increased EETs and HETEs may help to improve hypertension. The derivative of EETs was found to be antihypertensive, to protect vascular endothelial function, and to inhibit renal tubular sodium channel [i.e., epithelial sodium channel (ENaC)] in angiotensin II-dependent hypertension (Hye Khan et al., 2014). Besides, EETs are the potent endothelium-derived vasodilators that modulate vascular tone through the enhancement of Ca^2+^-activated K^+^ channels in vascular smooth muscle (Baron et al., 1997). In addition, 16-HETE and 18-HETE were shown to produce renal vasodilation, and they exhibited the inhibition of proximal tubule ATPase activity. Subterminal HETEs may participate in renal mechanisms affecting vasomotion (Carroll et al., 1996). Zhang et al. (2005) reported that the levels of 18-HETE were significantly decreased in renal interlobar arteries of spontaneously hypertensive rats.

Moreover, we demonstrated hyperlipidemia with significantly increased PGJ2 level in high iodide intake–induced hypothyroidism and found significant correlations between 4-HDoHE, 8-HDoHE, TXB2, 5,6-EET, 11,12-EET, 14,15-EET, 16-HETE, 15-oxo-ETE, and dyslipidemia. It was reported that the causes of hyperlipidemia in hypothyroidism are the decreased expression of hepatic LDL receptors, which reduces cholesterol clearance, and the reduced activity of cholesterol-α-monooxygenase, an enzyme that breaks down cholesterol (Canaris et al., 2000; Jabbar et al., 2017). PGJ2 metabolized further to yield ^Δ12^-PGJ2 and 15-deoxy-^Δ12, 14^-PGJ2 (15d-PGJ2) (Abdelrahman et al., 2004). PGJ2 and PGD2 exhibited an effect similar to 15d-PGJ2 (Kasai et al., 2000). 15d-PGJ2 is a natural ligand for peroxisome proliferator-activated receptor γ (PPARγ), which functions as a transcriptional regulator of genes linked to lipid metabolism (Ricote et al., 1999). There are findings which indicate that 15d-PGJ2 may stimulate the production of TG (Kasai et al., 2000). In this study, high iodide intake–induced hypothyroidism associated with hyperlipidemia was significantly improved after the treatment of iodide intake adjustment + 1,25(OH)_2_D_3_ supplementation, with significantly increased EETs (i.e., 5,6-EET, 8,9-EET, 11,12-EET, and 14,15-EET), 5-oxo-ETE, and 15-oxo-ETE. It was reported that 5,6-EET, 8,9-EET, 11,12-EET, and 14,15-EET can be metabolized by cytochrome P450 2J2 (CYP2J2). Zhang S. S. et al. (2015) reported that endothelial-specific CYP2J2 overexpression can decrease TG, TC, and FFA levels in the liver of hyperlipidemic mice by enhanced FFA β-oxidation, which was mediated by the AMPK and PPARα pathway. 5-oxo-ETE and 15-oxo-ETE are the metabolites of 5-HETE and 15-HETE, respectively. Grzesiak et al. reported that TG was correlated with 5-HETE and 15-HETE, TC was correlated with 15-HETE in patients with both benign prostatic hyperplasia (BPH) and metabolic syndrome (MetS), and lipid mediators of inflammation, which influence the levels of biochemical parameters, may contribute to the mechanism (Grzesiak et al., 2019).

Furthermore, our results indicated that PGB2, PGE2, 16-HETE, 18-HETE, 8,9-DHET, and 7-HDoHE were correlated with the function of the thyroid. In addition, the significantly increased TSH level with increased PGB2 and the significantly decreased VD3 level with increased PGB2 and PGJ2 were detected in high iodide intake–induced hypothyroidism. PGB2 is the metabolite of PGE2 (Coras et al., 2021), and PGJ2 is formed by dehydration within the cyclopentenone ring of PGD2 (Abdelrahman et al., 2004). Tahara et al. (1991) reported that TSH stimulates the production of PGD2 and PGE2 in the Fischer rat thyroid follicular cell line (FRTL-5). Menon et al. (2019) reported that PGI2 analog was the mainstay of treatment for severe pulmonary arterial hypertension (PAH). PGs, in particular, prostacyclin, and their analogs cause a variety of side effects, such as hyperthyroidism, autoimmune goiter, Graves' disease, Hashimoto's disease, and thyrotoxicosis, in patients with PAH, and therapy with medications targeting the prostacyclin pathway is a potential risk factor for the development of symptomatic thyroid disease. The activation of PG receptors in the thyroid gland leads to the production of cyclic AMP (cAMP), which, in turn, stimulates the production of thyroid hormone and may contribute to the mechanism (Menon et al., 2019). The elevated levels of VD3 and PGE2 were detected. Although there was no significant change detected for PGB2, a downward trend was apparent after the treatment of iodide intake adjustment + 1,25(OH)_2_D_3_ supplementation. Liu et al. (2014) reported that all three forms of vitamin D reduced the production of PGE2 by stimulating HPGD, an enzyme that degrades PGE2. The onset of autoimmune thyroid disease with vitamin D deficiency is very common (Clinckspoor et al., 2012). Some studies indicated that vitamin D deficiency is a predisposing condition for autoimmune diseases (Peterlik et al., 2009). PGE2 can serve both pro-inflammatory and anti-inflammatory functions (Frolov et al., 2013). Qian et al. (2011) reported that PGE2 negatively regulates inflammation by inhibiting C-C chemokine ligand 5 (CCL5) expression in activated macrophages. Loynes et al. (2018) illustrated that the production of PGE2 at sites of tissue injury promotes an anti-inflammatory neutrophil phenotype and determines the outcome of inflammation resolution *in vivo*.

However, the studies to determine the mechanism of fatty acids in hypothyroidism and its complications are largely unknown. It is reported that the Ca/phosphoinositide/AA signal system is important to both the function and the growth of FRTL-5 rat thyroid cells and to the action of both TSH and alpha-1 adrenergic agents. This action was accompanied by the increases in cytosolic Ca^++^, the release of AA from the cells, and the action of AA metabolites in processes important to the formation and growth of thyroid hormone (Tahara et al., 1989). Coria et al. reported that thyroid hormones are the important regulators of lipid metabolism, and hypothyroidism may reduce the relative contents of AA (Coria et al., 2012). In PTU-induced hypothyroidism, all the enzyme activities involved in the biosynthesis of fatty acids (i.e., acetyl-CoA carboxylase, fatty acid synthetase, and microsomal chain elongation and desaturation reactions) are strongly reduced after 3 days of drug administration.

Most studies on thyroid cancer were focused on AA. The AA is the precursor of PGs, which is a class of oncogenic lipid signaling molecules. Sun et al. reported that AA is a biomarker of papillary thyroid cancer (PTC). AA was significantly increased in PTC tissues from an iodine excess area compared with tissues from an iodine adequate area. The high levels of iodine may inhibit the activity of metabolic enzymes, such as COX, LOX, and LYP450, which in turn leads to a significant decrease in the synthesis of PGs (Sun et al., 2021). While AA was significantly decreased in PTC tissue compared with para-PTC tissue in both tissues from iodine adequate area and iodine excess area, a decrease in AA could be explained by the increased generation of PGs in PTC (Sun et al., 2021). Chen et al. reported that the relative levels of AA decreased in PTC. PG-endoperoxide synthase 2 (PTGS2; also known as COX-2) (Kunzmann et al., 2013) catalyzes the conversion of AA to PG, the mRNA level of PTGS2 was increased in PTC, and an increased consumption of AA was observed, which forms the oncogenic lipid in PTC (Chen et al., 2015). Krawczyk-Rusiecka et al. (2014) reported that there was a significantly higher expression level of the COX-2 gene in the PTC group, in comparison with Hashimoto's thyroiditis (HT) and non-toxic nodular goiter (NNG) groups. Reyes et al. (2019) also reported that an elevated arachidonate 5-lipoxygenase (ALOX5) was detected in patients with PTC. Kummer et al. (2012) reported that ALOX5 protein and mRNA were upregulated in PTC and that ALOX5 expression positively correlated with invasive tumor histopathology. Kim et al. (2003) reported that the levels of AA and DHA were significantly decreased in the urine profiles of the patients with thyroid cancer compared with normal female subjects, and the decreased level of glucocorticoids induced from the decreasing urinary concentration of DHA may play an important role in thyroid cancer. Berg et al. (1994) found that the high serum levels of AA and DHA provide a protective effect, and the low serum levels provide the risk of developing thyroid cancer. AA and DHA possibly may prevent thyroid cancer by reducing the estrogen receptor contents in thyroid tissues. Ji et al. (2012) reported that COX-2 expressions were stronger in thyroid carcinoma than in thyroid adenomas and normal tissues and that the COX-2 expressions in thyroid carcinoma were correlated with the tumor type and tumor-node-metastasis (TNM) stage. They also suggested that the expression of COX-2 may promote angiogenesis, infiltration, and metastasis of of thyroid carcinoma. Puxeddu et al. (2003) reported that COX-2 is overexpressed in thyroid malignancies compared with benign nodules and normal thyroid tissues. Alexanian et al. (2012) reported that the expression of CYP4A/4F genes was markedly elevated in the samples of thyroid cancer in comparison with matched normal tissues.

This study has some limitations. Further investigations on the measurements of echocardiography, blood pressure, and serum fatty acids in other time points and also on the expression of COXs, CYP450, and LOX need to be validated in our future research.

## Conclusion

The increased PGs (PGB2 and PGJ2) and decreased 8,9-DHET levels might take part in the progression of cardiac dysfunction, hypertension, and dyslipidemia in high iodide intake–induced hypothyroidism. Significantly increased EETs (i.e., 5,6-EET, 8,9-EET, 11,12-EET, and 14,15-EET) and HETEs (5-oxo-ETE, 15-oxo-ETE, 16-HETE, and 18-HETE) might represent the key regulators of these complications after iodide intake adjustment + 1,25(OH)_2_D_3_ supplementation. This novel aspect of fatty acids may provide new insights into high iodide intake–induced hypothyroidism and its complications.

## Data Availability Statement

The original contributions generated for the study are included in the article, further inquiries can be directed to the corresponding author.

## Ethics Statement

The animal study was reviewed and approved by the Institutional Animal Care and Use Committee of Tianjin Medical University (no. TMUaMEC 2016054).

## Author Contributions

QL, YZ, and HZ performed the experiments and analyzed the data. QL, HZ, and XY wrote the manuscript. XY designed the theme and experimental methods of the study. All authors carried out this research.

## Conflict of Interest

The authors declare that the research was conducted in the absence of any commercial or financial relationships that could be construed as a potential conflict of interest.

## Publisher's Note

All claims expressed in this article are solely those of the authors and do not necessarily represent those of their affiliated organizations, or those of the publisher, the editors and the reviewers. Any product that may be evaluated in this article, or claim that may be made by its manufacturer, is not guaranteed or endorsed by the publisher.
